# Oocyte selection: a tale of individualism, dominance and sacrifice

**DOI:** 10.1038/s44319-025-00665-5

**Published:** 2025-12-08

**Authors:** Katja Wassmann

**Affiliations:** Université Paris Cité, CNRS, Institut Jacques Monod, 75013 Paris, France

**Keywords:** Autophagy & Cell Death, Development

## Abstract

New research in *EMBO Reports* reveals a phagocytosis-driven mechanism of oocyte selection in mouse.

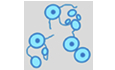

## Teaser

Oocyte development is a fierce competitive process in which only a small fraction of germ cells survives massive waves of cell death to become a fertilizable egg. Using an ex vivo 4D imaging system, Zhang and colleagues discovered that individual oocytes actively move, extend filopodia-like protrusions, and engulf the debris of their dying neighbors to fuel their own growth (Zhang et al, 2025a). These findings reveal a previously unrecognized, phagocytosis-driven mechanism of oocyte selection that shapes the ovarian reserve.

Oocytes are among the longest-lived cells in the mammalian body (Zhang and Liu, 2015). They are set aside before the birth of the female and remain dormant after completing meiotic recombination. In humans, they wait until they are stimulated to grow and—usually one by one—enter the meiotic divisions in adulthood, preparing them for fertilization. An oocyte that has reached this final step of female gametogenesis and becomes a haploid egg has survived several massive waves of selection: from around 5–7 million oogonia initiating meiosis, only 1–2 million are present at birth, and of these, only 250,000 to 500,000 remain at the onset of puberty (Zhang et al, 2025b). Ultimately, only ~400 oocytes are ovulated during the reproductive years, until the oocyte pool is depleted and menopause occurs (Gruhn and Hoffmann, 2022). How a given oocyte survives these extensive waves of oocyte death, and why so many oocytes are produced in the first place, remain open questions. It has been proposed that oocyte overproduction serves as some kind of quality control mechanism and only the best quality oocytes are used. Additionally, oocytes that have not been selected may contribute to the survival of selected oocytes (Tilly, 2001). A deeper understanding of the underlying molecular mechanisms is needed to find answers to these questions; however, studies of early oocyte loss and selection in mammals have been hindered by major technical challenges, as they require tracing individual oocytes during early ovarian development.

Early oocyte development takes place within a structure called the germline cyst, formed by primordial germ cells that have migrated to the gonad at embryonic day 10.5 in the mouse. At this stage, up to 30 germ cells are found per cyst, interconnected through intercellular bridges. Shortly after birth (postnatal day 4), only ~6 primary oocytes remain per cyst due to the loss of the others. Importantly, oocytes that are eliminated contribute organelles and cytoplasm to the surviving oocytes through the intercellular bridges, and this transfer contributes to the formation of the so-named Balbiani body, a structure enriched in organelles, proteins, and mRNAs, and specific to oocytes (Ikami et al, 2017; Niu and Spradling, 2022). It has been proposed that the number of intercellular bridges an oocyte forms with its neighbors determines whether it will survive. Stable intercellular bridge formation depends on Tex14 (Testis-expressed 14), a protein that blocks cytokinesis. However, even though Tex14-deficient female mice have fewer oocytes and lack intercellular bridges, they remain fertile, indicating that these bridges are not essential for oocyte development (Greenbaum et al, 2009; Spradling et al, 2022). This raises the possibility that additional mechanisms allow sacrificed oocytes to transfer cytoplasm and organelles to their surviving neighbors.

**Figure 1:**
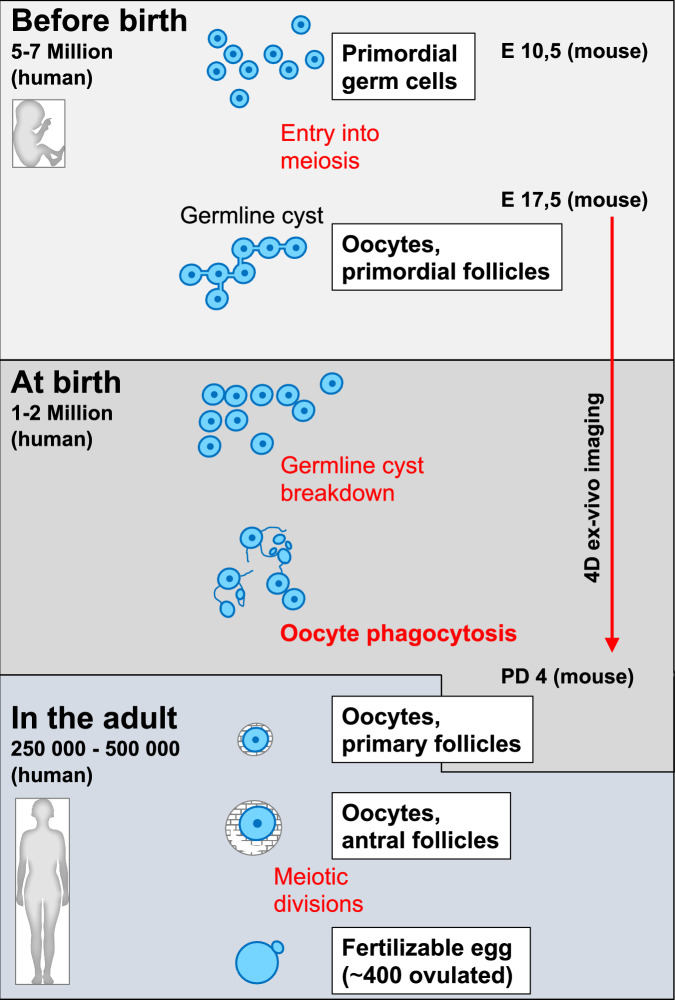
Simplified scheme of oogenesis in mammals. See text for details. On the left, the numbers indicate remaining oocytes derived from 5–7 million primordial germ cells. On the right, the corresponding days of development in the mouse are listed (E: embryonic day, PD: postnatal day). The red arrow shows the timeframe analyzed by ex vivo 4D imaging, revealing oocyte phagocytosis as a new mechanism to build the female reproductive reserve, at least in the mouse. Text in bold and red indicates the cyst-independent phagocytosis described in the present study.

The study by Zhang and colleagues in this issue of Embo Reports addresses this question. The authors hypothesized that oocyte selection around birth is a dynamic process that cannot be understood without following the fate of individual cells through long-term live imaging. In a technical tour-de-force, the authors established a 4D imaging system to monitor germ cell development in fetal ovaries cultured in vitro. Movements of individual cells were recorded in 3D for several days, starting at embryonic day 17.5, when germ cells have entered meiosis and can therefore be considered oocytes. Oocyte membranes were labeled with an oocyte-specific marker, enabling the authors to distinguish oocytes from other cells in the ovary and to track changes in oocyte morphology. Notably, this imaging period covers the developmental window in which major oocyte selection occurs (Fig. 1) (Pepling, 2006). Transplantation experiments after the imaging period showed that the oocytes selected in these ex vivo cultures were capable of normal growth, ovulation, and fertilization.

Several key observations emerged from this first dynamic analysis of the selection process. Unexpectedly, oocytes behaved as individual, actively motile cells within the cyst. Contacts between oocytes were only transient, and each oocyte interacted with several distinct neighbors during the imaging period. Retrospective analysis revealed that surviving oocytes formed highly dynamic filopodia-like structures. Oocytes that did not survive frequently broke down and appeared as cellular debris, which the authors termed oocyte debris. Crucially, surviving oocytes extended filopodia-like protrusions to engulf this oocyte debris. By randomly labeling oocyte cytoplasm with either CFP or RFP, the authors observed cytoplasmic mixing in some surviving oocytes, demonstrating that they had incorporated material from another oocyte. Single-cell RNA sequencing of oocytes isolated one day after birth and thus at the peak time of oocyte selection revealed distinct transcriptional signatures: one group displayed activation of cell-death and autophagy pathways, while another expressed genes involved in filopodia formation and genes characteristic of follicle-associated oocytes. These findings indicate that oocyte fate has been determined by this stage and oocytes express either genes required for their survival (filipodia, …) or associated with sacrifice (cell death pathway and autophagy genes).

In ex vivo culture, engulfment of neighboring oocytes correlated with increased oocyte volume. Pharmacological inhibition of phagocytosis or filopodia formation prevented the increase in oocyte volume and mitochondrial content in the selected oocytes. But how important is debris engulfment for generating high-quality oocytes in vivo? Using insights from the ex vivo imaging, combined with a whole-mount transparent imaging system as well as transmission electron microscopy, the authors identified filopodia-like structures and oocyte debris in intact ovaries. Crucially, they also observed engulfment events in freshly isolated ovaries. To test the functional relevance, they inhibited autophagy in ex vivo cultures followed by allo-transplantation. With the caveat that addition of these inhibitors affects overall cell survival (since autophagy is a basal, ongoing process in cells), their results are nevertheless conistent with phagocytosis being generally required for reproductive success, in agreement with an essential role for engulfment of oocyte debris during oocyte selection.

In conclusion, this work provides exciting new insights into the dynamics of oocyte selection using a sophisticated ex vivo 4D imaging platform. In the future, this experimental system will enable researchers to dissect the molecular mechanisms of phagocytosis and filopodia formation during oocyte selection, shedding light on how a single oocyte emerges as the winner at the expense of its neighbors.
